# The Cytodiagnosis of Amelanotic Primary Signet Ring Cell Melanoma at the Unusual Site of the Axilla: A Case Report of a Rare Entity

**DOI:** 10.7759/cureus.48714

**Published:** 2023-11-13

**Authors:** Kaustubh Kharche, Arvind Bhake

**Affiliations:** 1 Department of Pathology, Jawaharlal Nehru Medical College, Datta Meghe Institute of Higher Education and Research, Wardha, IND

**Keywords:** amelanotic primary signet ring cell melanoma, immunohistochemistry(ihc), fine needle aspiration cytology (fnac), signet ring cell melanoma, amelanotic melanoma

## Abstract

Amelanotic primary signet ring cell melanoma is a rare variant of cutaneous malignant melanoma. The diagnosis of amelanotic primary signet ring cell melanoma is rarely made based on cytological examinations. Most of the cases reported in the routine practice involve metastatic lesions of known cutaneous amelanotic primary signet ring cell melanoma. The diagnosis of amelanotic primary signet ring cell melanoma on fine needle aspiration cytology (FNAC) is scarce. We present one such extremely rare diagnosis of amelanotic primary signet ring cell melanoma at the unusual site of the axilla, which was established on FNAC. We also discuss its histological differential diagnosis and confirmative immunohistochemistry (IHC).

## Introduction

Malignant melanomas are associated with histologically diverse variants [1,2]. One of its extremely rare variants is signet ring cell melanoma. Signet ring cell melanoma is reported to constitute about 0.5% of all malignant melanoma cases [3]. While it usually occurs on the skin and mucosa, it is also reported at rare locations of anorectum [4] and gastroesophageal junction [5]. However, the diagnosis of primary cutaneous signet ring cell melanoma on fine needle aspiration cytology (FNAC) is rarely reported. One case report in the literature has described lymph node metastasis of signet ring cell melanoma on FNAC [6].

The diagnosis of signet ring cell melanoma has significant clinical implications as these tumors are not only locally aggressive but also show augmented metastasis [1]. There are scarce data in the literature about the cytodiagnosis of signet ring cell melanoma by FNAC. Mostly, the cytodiagnosis of signet ring cell melanoma reported on FNAC involves metastatic lesions in known patients with primary signet ring cell melanomas of other sites [6,7]. A brief report has described the potential diagnostic pitfalls and mimicking of signet ring cell melanoma as signet ring cell lymphoma, liposarcoma, and epithelioid mesenchymal tumors [6,7].

We discuss a case involving an extremely rare cytodiagnosis of amelanotic primary signet ring cell melanoma in the axilla of a 67-year-old male patient; we describe its cytomorphological features, histomorphology of biopsy, and immunohistochemistry (IHC).

## Case presentation

**Figure 1:**
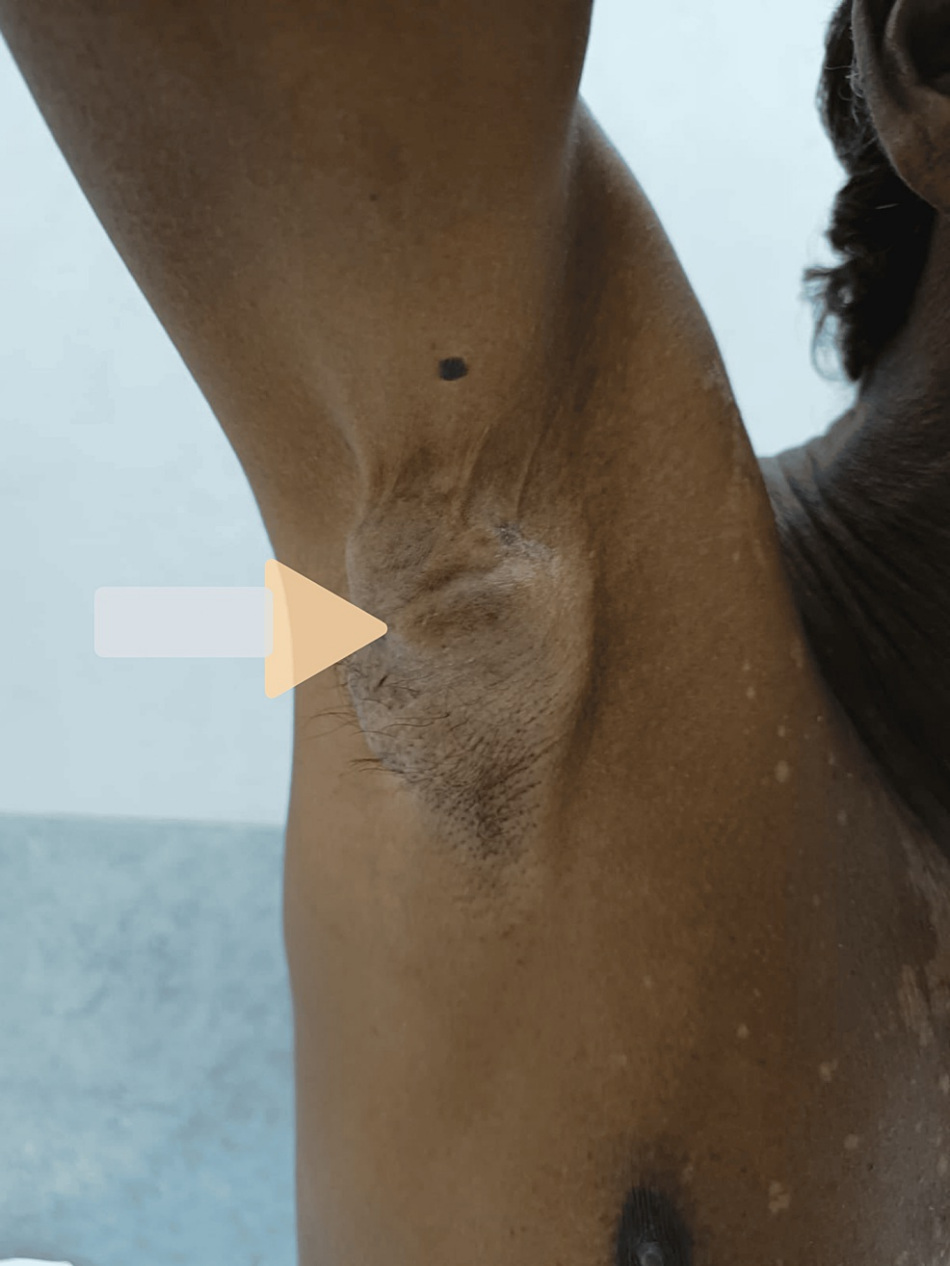
Clinical photograph of axillary swelling A swelling of 6 x 4 x 3 cm in size with hyperpigmentation in the center and involvement of the skin is demonstrated by stretching of the arm

A 67-year-old male presented to the outpatient section of the Department of Surgery of the University-attached Acharya Vinoba Bhave Rural Hospital complaining of swelling at the right-sided axilla. The patient had first noticed the swelling two years back and it had been progressively increasing in size. The swelling was 6 x 4 x 3 cm in size and located in the pit of the axilla. The skin over the swelling showed hyperpigmentation in the center surrounded by a collar of hypopigmented skin. The skin in the rest of the axilla appeared normal. There was a small mole on the skin in the vicinity of the swelling. The skin over the swelling was not free and lifting of the arm led to stretching of the skin around the swelling (Figure 1).

The swelling was firm to palpate and was not fixed to the underlying structure or muscles, and it was immovable under the skin. However, restricted mobility to the lateral side underneath the swelling was observed as it did not involve the structures below it. The patient was advised to undergo FNAC with the clinical diagnosis of malignant lymph node or tubercular lymphadenitis with periadenitis involving the skin. The examination of the ipsilateral, contralateral breast, and chest wall was normal. There were a few hypopigmented patches on the skin of the chest wall, back, neck, and shoulder. The general and systemic examination revealed no other primary tumor at other body sites.

The patient underwent an X-ray of the chest, which showed a soft tissue shadow in the right axilla. The thoracic cage and lung parenchyma appeared normal. The patient was then referred for FNAC under ultrasonographic (USG) guidance. Meanwhile, he underwent complete blood counts (CBC) and basic biochemistry as part of the required preoperative investigations. These investigations were within normal limits except for lower hemoglobin values. FNAC was carried out under USG guidance with the patient's consent. The material of FNAC was spread on glass smears which were dry-fixed as well as wet-fixed in 95% ethyl alcohol. The part of the material of FNAC was processed for cell block studies to obtain the paraffin section. These sections were later stained with hematoxylin and eosin stains (H and E).

The dry-fixed smears of FNAC underwent May Grunwald-Giemsa (MGG) stain and wet-fixed smears underwent Papanicolaou (PAP) stain and hematoxylin and eosin staining. The cytomorphology was diagnostically assessed in accordance with standard textbooks and references. The smears were cellular. The characteristic morphological features involved the population of epithelial cells. The smears showed multiple monolayered cell sheets and rare attempted pseudoglandular structures of intermediate-sized nonkeratinizing cells with reduced cohesion. In many places, these cells were isolated and dispersed. These cells carried enlarged hyperchromatic nuclei with prominent nucleoli in many as well as mild pleomorphism. The cytoplasm of the cell was modest and a few cells showed dusting of cytoplasm by brown colored pigment on PAP stain.

**Figure 2:**
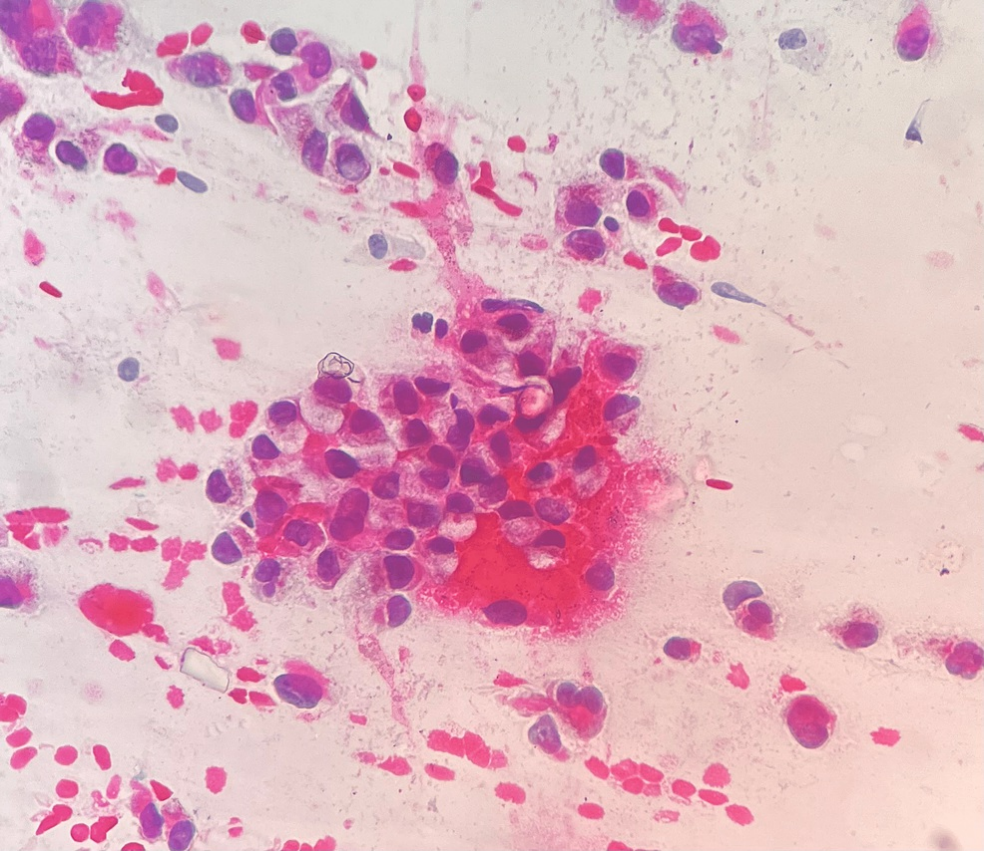
Photomicrograph: signet ring cell melanoma (FNAC, PAP stain, 40x) - image 1 The smear shows signet ring cells in poorly cohesive groups and accompanying isolated populations of malignant melanocytes FNAC: fine needle aspiration cytology

**Figure 3:**
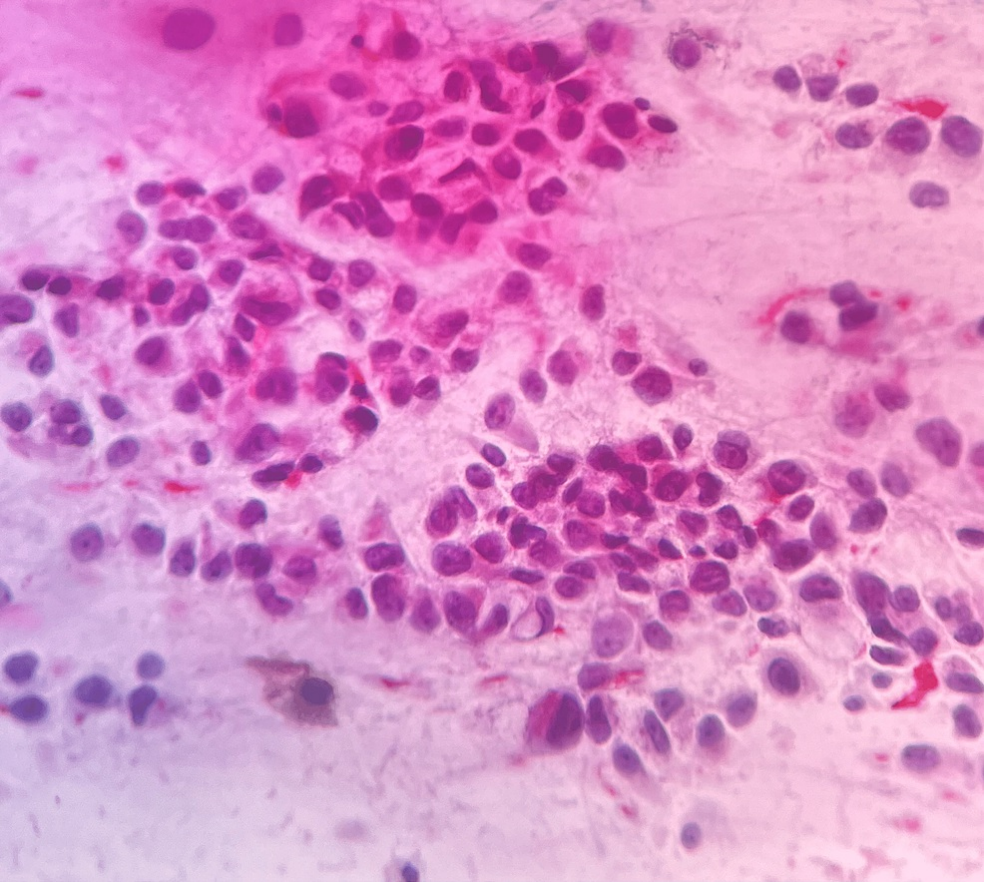
Photomicrograph: signet ring cell melanoma (FNAC, PAP stain, 40x) - image 2 The smear shows chiefly dissociated cell population, few of which show signetoid appearance, and rare malignant melanocytes show brown fine discoloration of cytoplasm FNAC: fine needle aspiration cytology

Rare mucinous appearing large vacuoles were seen in the cytoplasm with eccentric nuclei symbolizing signet ring cell morphology, and very rare melanin-containing macrophage was evident. The background showed rare binucleate cells and the striking presence of myxomucinous material with spindled stromal cell and endothelial cell loops. A few of these cell groups were seen to lie within myxomucinous material (Figure 2). The diagnosis of amelanotic signet ring cell melanoma was considered as the smears showed myxomucinous material (Figure 3). It was considered an extracellular change.

**Figure 4:**
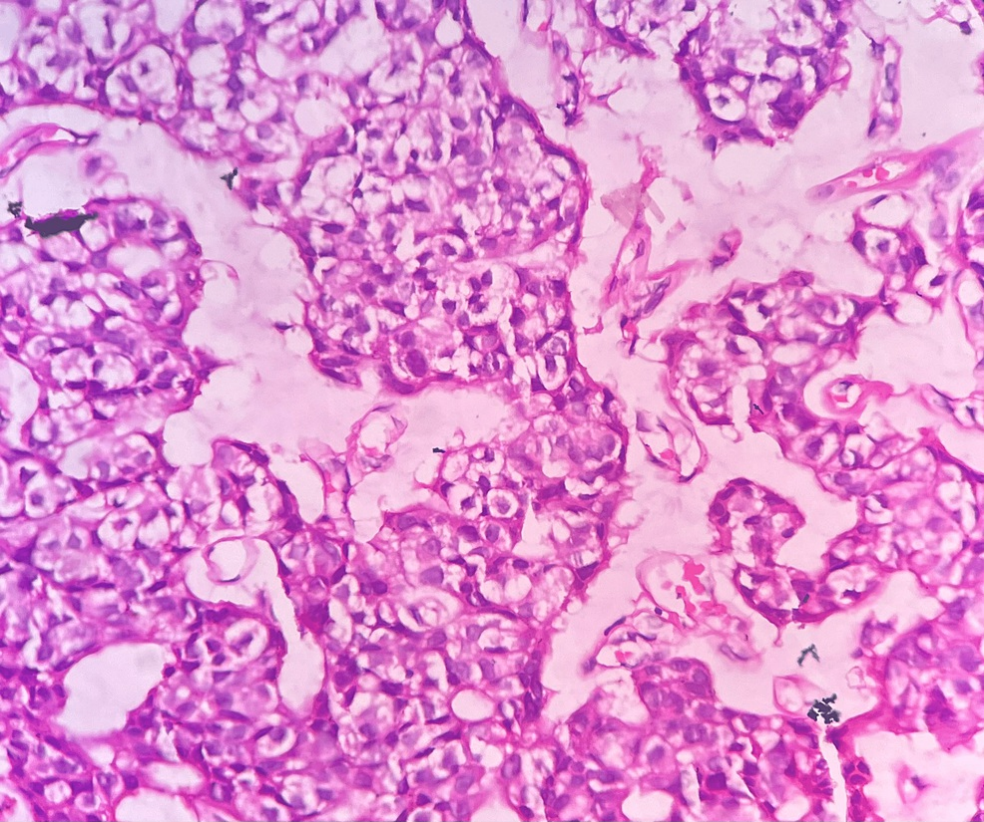
Photomicrograph: signet ring cell melanoma (histopathology, H and E stain, 40x) - image 1 The section shows signet ring cells placed in lobulated groups and a rare pseudoglandular pattern with myxomucinous material around the cell groups

**Figure 5:**
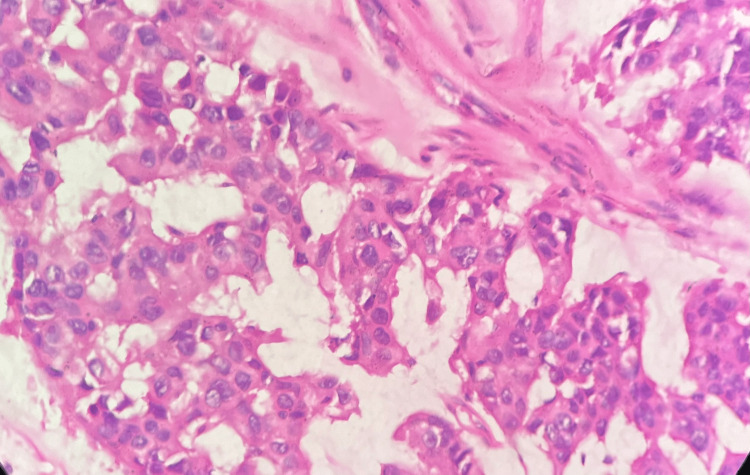
Photomicrograph: signet ring cell melanoma (histopathology, H and E stain, 40x) - image 2 The section shows malignant melanocytes placed in cords and small solid groups mostly with amelanotic cytoplasm and rare signet ring cytoplasm. The area between the cellular components shows mucomyxoid material

The cell block material showed features similar to cytology preparations. The signet ring cells were seen to be distributed among the cells of non-signet ring cell morphology (Figure 4). The patient underwent surgical excision of the tumor. The histology of the tumor was reported as primary mucinous adenocarcinoma with signet ring cells with another differential of signet ring cell melanoma with myxomucinous degeneration (Figure 5).

**Figure 6:**
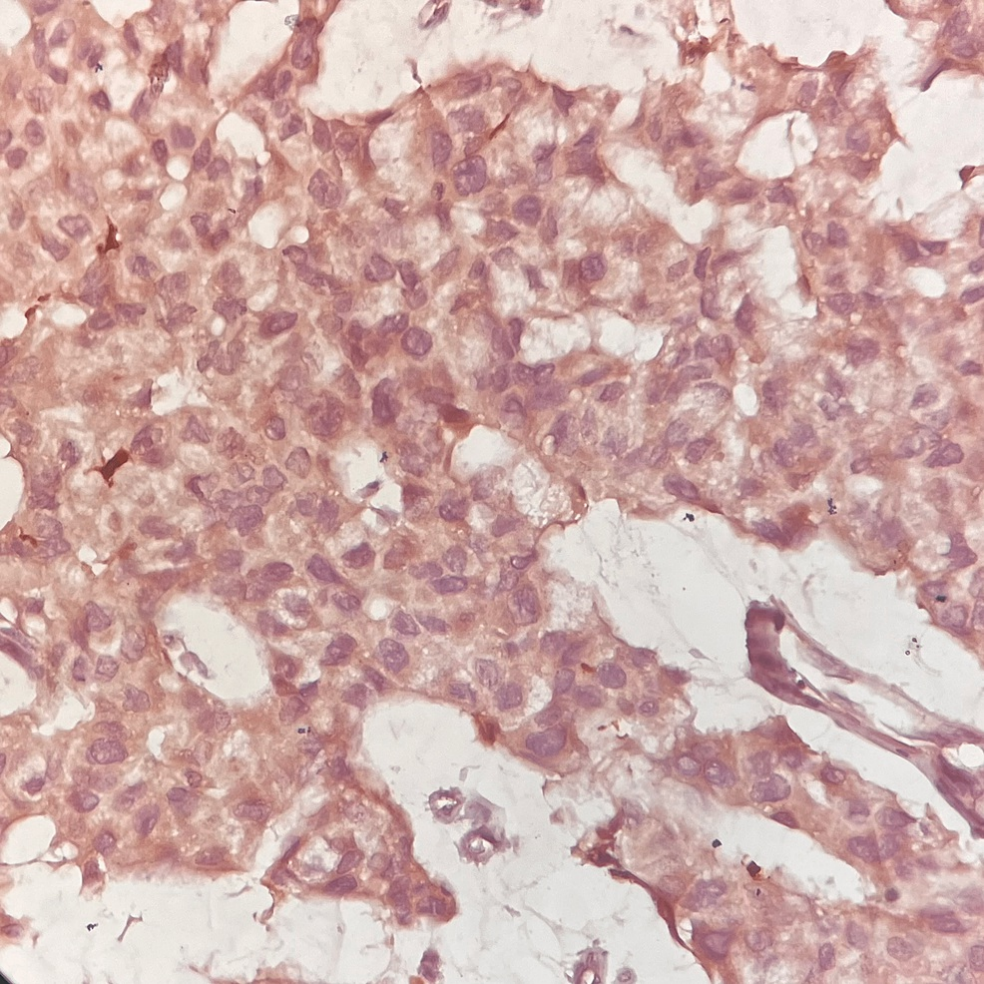
Immunohistochemistry (IHC) for S100 protein (IHC, 40x) - image 1 The section shows cytoplasmic brown granularity of positive immunostaining including signet cells for S100 protein

**Figure 7:**
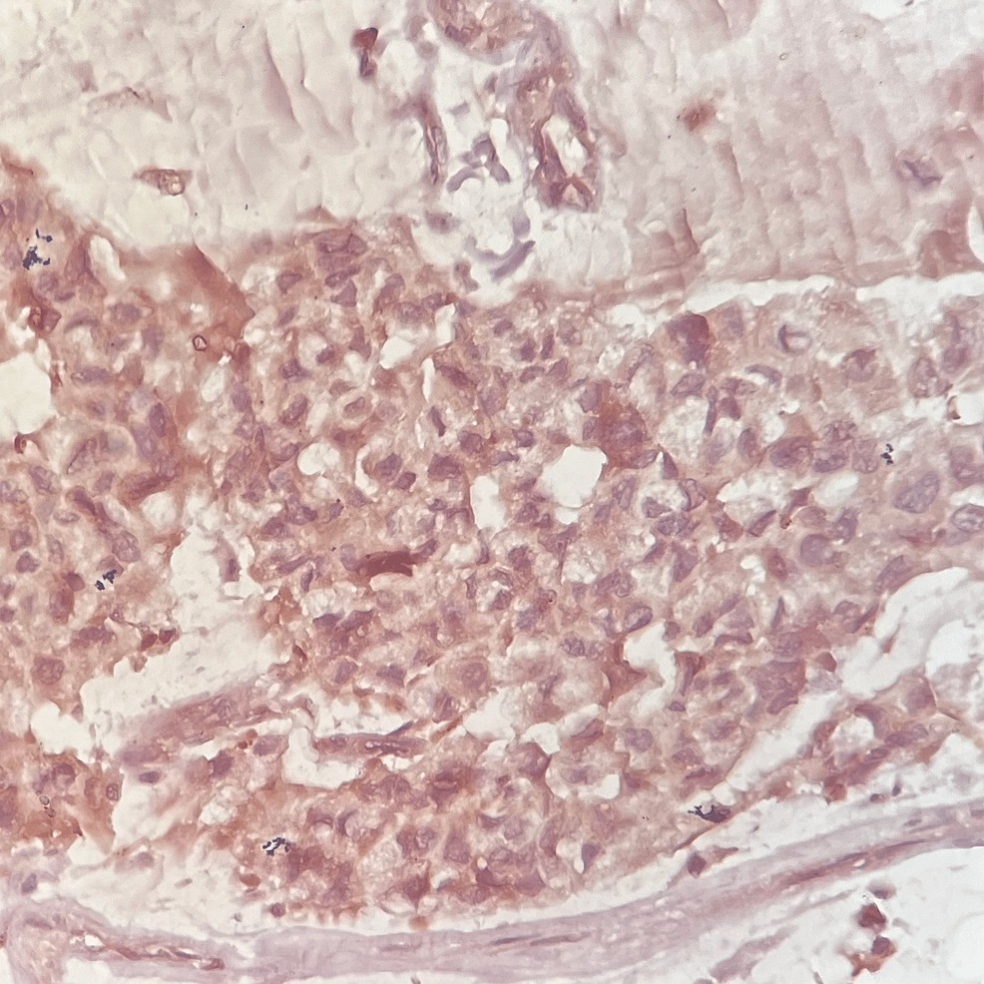
Immunohistochemistry (IHC) for S100 protein (IHC, 40x) - image 2 The section shows background brown staining, which reveals positive immunostaining for S100 protein

The paraffin blocks of the tumor underwent IHC for S100 protein. The section showed brown granular cytoplasmic positivity for S100 IHC (Figure 6). The signet ring cells showed peripheral brown discoloration for S100 protein, as seen in Figure 7.

## Discussion

Primary signet ring cell melanoma is an extremely rare variant of malignant melanoma [2]. The morphological diagnosis of the condition is based on the signetoid morphology of malignant melanocytes [2-4]. Therefore, signet ring cell melanoma is often associated with a potential diagnostic pitfall [7]. The elements of differential diagnosis of the condition in the skin are few but still worthy of consideration. One of the common differentials entails the cutaneous metastasis of signet ring cell carcinoma arising in the gastrointestinal tract [5]. Clinicians also need to exclude signet ring cell non-Hodgkin’s lymphoma, pleomorphic liposarcoma, and primary cutaneous mucinous adenocarcinoma [4-6].

The morphological differences that help to differentiate the aforementioned entities are slender and thin. The supportive histomorphological evidence for a primary signet ring cell melanoma involves the presence of non-signet classical malignant melanocytes. The majority of signet ring cell melanomas are amelanotic, which compounds the histomorphological confusion [6,7]. The IHC for S-100, HMB-45, and Melan-A confirms the diagnosis of signet ring cell melanoma. These markers are negative in the cells of signet ring cell carcinoma of signet ring cell non-Hodgkin’s lymphoma.

The present case report is unique for several reasons. It was the first such case diagnosed on FNAC. The electronic literature search regarding the diagnosis of amelanotic primary signet ring cell melanoma did not elicit much data, except for one report about the cytodiagnosis of metastasis of signet ring cell melanoma in a lymph node on FNAC in a case with known primary [6]. The description of the cells that this report dispenses is similar to the appearance of the cells seen in the present case.

One study has described the FNAC of signet ring cells in various types of breast carcinoma in 11 cases, but it did not mention signet ring cell melanoma. The study reported the presence of signet ring cell formations in many cutaneous neoplasms on biopsy material [7]. it discussed a rare case of amelanotic signet ring cell melanoma presenting as a breast lump [7]. However, this report does not provide a description of the cytology of the lump. The axillary location of signet ring cell melanoma too is very uncommon.

The present case report highlights the implications of observing signet ring cells in cytological preparations from a lump in the axilla. The female axilla may harbor extramammary or ectopic mammary tissue. A similar such appearance on histomorphology may raise the possibility of mucinous signet ring cell carcinoma of the breast in axillary mammary inclusions. However, our case involved a 67-year-old male. The possibility of axillary mammary inclusions harboring signet ring cell carcinoma in males is likely to be minuscule. The patient in the present case report did not harbor any primary epithelial neoplasm as per the clinico-radiological examination. Therefore, the possibility of it being a metastasis does not arise. This is an additional unusual clinical finding with regard to signet ring cell melanoma.

## Conclusions

This case report highlights the role of FNAC as a mandatory preoperative diagnostic intervention in cases involving patients such as ours. It provides insight into preoperative diagnosis and auxiliary investigations are called for. The authors do not claim that this is the first report involving the diagnosis of an unusual variant of signet ring cell melanoma at an equally unusual site in a male axilla. Nevertheless, this case is still worth sharing as it can serve as a unique learning experience regarding the cytomorphological appearances of amelanotic primary signet ring cell melanoma.
